# Brain region’s relative proximity as marker for Alzheimer’s disease based on structural MRI

**DOI:** 10.1186/1471-2342-14-21

**Published:** 2014-06-02

**Authors:** Lene Lillemark, Lauge Sørensen, Akshay Pai, Erik B Dam, Mads Nielsen

**Affiliations:** 1 Department of Computer Science, University of Copenhagen, Universitetsparken 1, 2100 Copenhagen Ø, Denmark; 2 Biomediq, Fruebjergvej 3, 2100 Copenhagen Ø, Denmark

**Keywords:** Alzheimer’s disease, Mild cognitive impairment, Bio markers, MRI, Diagnosis and classification, Proximity

## Abstract

**Background:**

Alzheimer’s disease (AD) is a progressive, incurable neurodegenerative disease and the most common type of dementia. It cannot be prevented, cured or drastically slowed, even though AD research has increased in the past 5-10 years. Instead of focusing on the brain volume or on the single brain structures like hippocampus, this paper investigates the relationship and proximity between regions in the brain and uses this information as a novel way of classifying normal control (NC), mild cognitive impaired (MCI), and AD subjects.

**Methods:**

A longitudinal cohort of 528 subjects (170 NC, 240 MCI, and 114 AD) from ADNI at baseline and month 12 was studied. We investigated a marker based on Procrustes aligned center of masses and the percentile surface connectivity between regions. These markers were classified using a linear discriminant analysis in a cross validation setting and compared to whole brain and hippocampus volume.

**Results:**

We found that both our markers was able to significantly classify the subjects. The surface connectivity marker showed the best results with an area under the curve (AUC) at 0.877 (*p*<0.001), 0.784 (*p*<0.001), 0,766 (*p*<0.001) for NC-AD, NC-MCI, and MCI-AD, respectively, for the functional regions in the brain. The surface connectivity marker was able to classify MCI-converters with an AUC of 0.599 (*p*<0.05) for the 1-year period.

**Conclusion:**

Our results show that our relative proximity markers include more information than whole brain and hippocampus volume. Our results demonstrate that our proximity markers have the potential to assist in early diagnosis of AD.

## Background

Alzheimer’s Disease (AD) is the sixth-leading cause of death in the US and accounts for 50-56 *%* of the cases of diagnosed dementia [1]. AD is often diagnosed in people over 65 years but the onset for AD can occur much earlier. The population of aged 65+ years in the US is estimated to double by the year 2030 [1]. This means that the number of new and existing cases of AD will increase drastically as well. At the moment, no cure or treatment is found for AD which obviously makes it a growing problem [1].

The causes of AD are not fully clarified. Research indicates that accumulation of twisted protein fragments inside the nerve cells, neurofibrillary tangles, and toxic protein fragment, amyloid beta oligomers, are characteristics of AD [2,3]. The hippocampus, which is associated with memory, is particular vulnerable to damage at the earliest stages of AD [3,4]. Hippocampal brain changes, such as loss of thickness and volume in the medial temporal lobe, particular in the hippocampus, is thought to begin 7 years or more before AD symptoms, such as memory loss, appear [5-8].

The cognitive decline can be slowed when administered during the early stage of disease [9], and therefore early detection of brain changes is highly desirable, both to increase the quality of AD patient’s life but also for future developments in drug trials.

Structural magnetic resonance imaging (MRI) has shown great applicability to map how AD spreads in the living brain. Recent literature have studied the accuracy and reproducibility of MRI-derived measurements and found correlation with clinical measurements and positive prediction of future decline [10-12]. MRI is non invasive and is largely available in the clinical environment, and has therefore become a viable tool to monitor the progression of AD. Longitudinal measurement changes may be more objective, precise, and reproducible when they are measured from MRI compared to diagnostic image measurements from PET scanning or CSF measurement of amyloid and tau protein.

Different studies have focused on the hippocampus region and used atrophy scoring and shape analysis for detection of AD [13-16]. Also whole brain atrophy scoring have been extensively used for detection of AD [17,18]. The same techniques have been used to classify MCI-converters (MCI-c) from MCI-non-converters (MCI-nc), indicating that it is possible to make a prognosis of AD based on atrophy rates, shape analysis, region hippocampal shapes and machine learning techniques [10,19-24].

The general focus on studies from MRI have been on the atrophy rates for hippocampus or the whole brain, or the shape of hippocampus, but other brain structures, like amygdala, putamen, thalamus, and the ventricles have also shown relation to AD [25-27]. We want to include all of these structures in order to investigated the relationship and proximity between different regions in brain in hope to characterize how the brain develops and use this as a marker for AD. We believe that the relationship between the positions of different regions or the surface connectivity between the different regions in the brain can capture how the atrophy spreads. We have used a Procrustes marker that classified AD subjects based on the position of the center of mass of each region in a Procrustes aligned environment, and a surface connectivity marker that extracted the percentile surface connectivity between the individual regions. The Procrustes marker can capture how the regions move away or toward each other indicating how the volume loss is different across the brain. The surface connectivity marker can describe the individual volume loss of the regions and how they move apart due to for example the increase in ventricles and cerebrospinal fluid (CSF). These new markers could give a more detailed view of the AD progression and may be used in addition to the traditional morphometric markers. Our markers were used in three different groupings of the brain regions; a group of all Freesurfer segmented regions, a subset of the functional regions, and a subset of the small potato shaped regions (for example hippocampus and amygdala) to classify, using a linear discriminant analysis, NC, MCI, and AD. This was done in comparison to the whole brain volume and hippocampus volume. Potentially, this could lead to a fine-to-coarse scale from where one can study the progression of AD from the global brain scale down to the local scale of the shape and/or texture of the individual sub-regions.

## Methods

### ADNI brain MRI and preprocessing

Data was obtained from the Alzheimer’s Disease Neuroimaging Initiative (ADNI) database (http://adni.loni.usc.edu) [28]. The ADNI was launched in 2003 by the National Institute for Aging (NIA), the National Institute of Biochemical Imaging and Bioengineering (NIBIB), the Food and Drug Administration (FDA), private pharmaceutical companies and non-profit organizations as a *$* 60 million, 5-year public-private partnership. The primary goal of ADNI has been to test whether serial MRI, positron emission tomography PET, other biological markers, and clinical and neurophysiological assessments can be combined to measure the progression of MCI, and early AD. Determination of sensitive and specific markers of very early AD progression is intended to aid researchers and clinicians to develop new treatments and monitor their effectiveness, as well as lessen the time and cost of clinical trials. The Principal Investigator of this initiative is Michael W. Weiner, MD, VA Medical Center and University of California - San Francisco. ADNI is the result of efforts of many co-investigators from a broad range of academic institutions and private corporations, and subjects have been recruited from over 50 sites across the U.S. and Canada. The initial goal of ADNI was to recruit 800 subjects but ADNI has been followed by ADNI-GO and ADNI-2. To date these three protocols have recruited over 1500 adults, ages 55 to 90, to participate in the research, consisting of cognitively normal older individuals, people with early or late MCI, and people with early AD. The follow up duration of each group is specified in the protocols for ADNI-1, ADNI-2 and ADNI-GO. Subjects originally recruited for ADNI-1 and ADNI-GO had the option to be followed in ADNI-2. For up-to-date information, see http://www.adni-info.org.

Longitudinal brain T1 weighted MRI and associated data for the study population including age, gender, and diagnosis were downloaded from the ADNI database. All data in this paper were from ADNI-1. ADNI-1 was a five year study launched in 2004 to develop longitudinal outcome measures of Alzheimer’s progression using serial MRI, PET, biochemical changes in CSF, blood and urine, and cognitive and neuropsychological assessment acquired at multiple sites similar to typical clinical trials. All subjects underwent clinical and cognitive assessment at the time of scan acquisition. All AD subjects met NINCDS/ADRDA criteria for probable AD [29]. The study was conducted according to the Good Clinical Practice guidelines, the Declaration of Helsinki and U.S. 21 CFR Part-50 Protection of Human Subject, and Part 56-Institutional Review Boards. This study was approved by the Institutional Review Boards of all of the participating institutions and informed written consent was obtained from all participants at each site.

### MRI acquisition

High-Resolution structural brain MRI were acquired at 59 ADNI sites using 1.5 Tesla T1-weighted MRI scans using volumetric 3D MPRAGE or equivalent protocols with varying resolution; typically 1.25×1.25 mm in-plane spatial resolution and 1.2 mm thick sagital slices. The MPRAGE sequence was acquired twice for all subjects at each visit to improve the chance that at least one scan would be suitable for analysis. The image quality was graded qualitatively by ADNI investigators of the ADNI MRQ quality control center at the Mayo Clinic for artifacts and general image quality. Each scan was graded on several separate criteria: blurring/ghosting, flow artifacts, intensity a homogeneity, signal-to-noise ratio, susceptibility artifacts and gray-white/cerebrospinal fluid contrast. We have only used the MRI scan which was graded as the best scan for each subject. No other exclusion criteria based on image quality were applied. We have used the raw ADNI data.

### Participants

The criteria for inclusion were those defined in the ADNI protocol; normal control (NC) subjects had a mini mental state examamination score (MMSE) between 24 - 30, a clinical dementia rating (CDR) score of zero, they were non-depressed, non MCI, and non-demented. MCI had MMSE scores between 24-30, a memory complaint, had objective memory loss measured by education adjusted scores on Wechsler Memory Scale Logical Memory II [30], a CDR of 0.5, absence of significant levels of impairment in other cognitive domains. AD subjects had MMSE scores between 20-26, CDR of 0.5 or 1.0 and met NINCDS/ADRDA criteria for probable AD. We selected a subset of 528 participants in the ADNI study. We have chosen a training set of 101 subjects based on statistics and visual inspection in order to get representative data which also included the difficult images, e.g., with image noise, and huge deformation to allow validation of our methods on a hard data set, which makes significant results more plausible.

The remaining 427 were taken as ADNI-1 data set [31] minus the overlap with the 101 subjects selected for training. Our subset population included 174 NC (age at baseline (bl) 76.0 years (y) ±5.1 y, 89 males (M)/85 females (F), 240 MCI (age at bl 74,9 y ±7.0y, 159M/81F), and 114 AD subjects (age at bl 74 y ±7.3 y, 58M/56F). There was 4 NC, 21 MCI and 7 AD subjects in our study that was under 65 y. Even though there is evidence that the pathology is different in early-onset AD and late-onset AD we have included the subjects under 65 because they do not have verified early onset AD [32]. The demographic details of our training and testing subjects are shown in Table 1.

**Table 1:** The demographic details of our study population

**Group**	**Number**	**Age at bl (years)**	**Gender (M/F)**	**MMSE at bl**
NC training set	24	75.3 ± 4.4 [65.1−85.9]	14 M/10 F	29.3 ± 1.1 [26−30]
MCI training set	29	73.6 ± 7.3 [55.2−85.5]	19 M/10 F	27.2 ± 1.6 [24−30]
AD training set	48	74.8 ± 6.7 [62.5−87.9]	24 M/24 F	23.5 ± 1.9 [21−26]
NC	174	76.0 ± 5.1 [60.0−89.7]	89 M/85 F	29.2 ± 1.0 [25−30]
MCI	240	74,9 ± 7.0 [55.2−88.4]	159 M/81 F	27.1 ± 1.7 [24−30]
AD	114	74,7 ± 7.3 [56.5−89.2]	58 M/56 F	23.3 ± 1.9 [20−26]

MMSE = mini mental state examination score. Values are indicated as mean ± standard deviation [*range*]. There is 4 NC, 21 MCI and 7 AD subjects in our study that is under 65.

### Freesurfer segmentation

The segmentation of the regions was performed by static FreeSurfer [33] implemented on a Linux cluster with 24 cores with 18 GB RAM per CPU. Freesurfer is a set of software tools designed to study the cortical and subcortical anatomy of the brain. Freesurfer do an affine registration of the volumes with the Talairach atlas [34], a non-uniform intensity normalization (N3) [35] and a B1 bias field correction [36]. A skull stripping step was performed using a deformable template model. Voxels were then defined as white matter or not white matter based on intensities. Hereafter, cutting planes were used to separate the hemispheres, cerebellum and brain stem. A cortical and subcortical labeling was performed based on a transformation that maps the individual subjects into a probabilistic atlas. The atlas was build based on a training set where the subjects have been labeled by hand and currently consists of 39 subjects distributed in age and AD pathology (28 NC and 11 with questionable or probable AD) [37]. The classification of each point was achieved by finding the segmentation that maximized the probability of input given the prior probability from the training set in a iteratively manner.

### Grouping of the segmented regions

**Figure 1:**
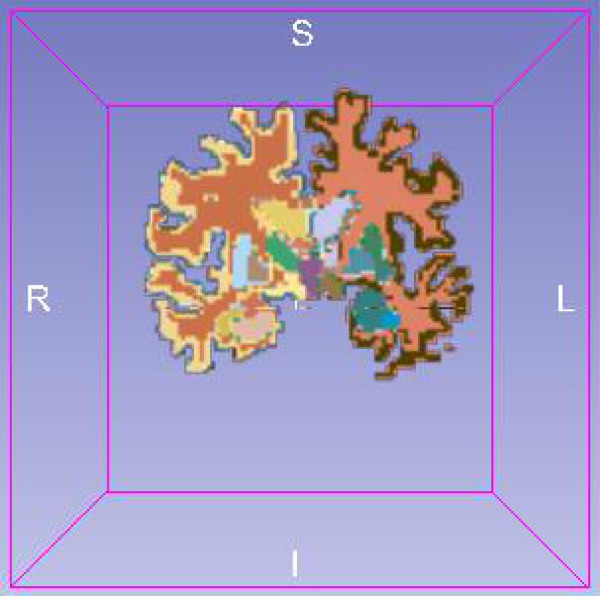
A slide of the segmented brain where the segmented regions have different colors.

The FreeSurfer segmentation provided 40 regions from which a visualization is shown in Figure 1. AD do not spread evenly across the brain and we are interested in capturing early signs of AD and the conversion from MCI to AD [3,25]. Therefore have we divided our regions into three groups; all, functional (func) and potato, described in Table 2. These groups are spread across the brain so we are not biasing toward anatomical placed groupings. The all group included the FreeSurfer segmented regions excluding left-vessel, right vessel and 5th ventricle because these regions were not segmented by FreeSurfer in all subjects. The functional group has excluded all non-function regions like CSF and hypointensities. The choroid plexus was included in the functional regions due to suggestions that the functionality is altered in the choroid plexus due to AD [38]. To get a even smaller subset, the potato group consisted of small potato shaped regions from a visual perspective where shape is clearly defined.

**Table 2:** The three different groups of the regions; all, functional and potato and the regions belonging to each group

All	CSF, 3rd-Ventricle, 4th-Ventricle, Brain-Stem, Optic-Chiasm
	WM-hypointensities, non-WM-hypointensities, left and right
	cerebral white matter, cerebral cortex, lateral ventricle, inf
	lateral ventricle, cerebellum white matter, cerebellum cortex,
	thalamus, caudate, putamen, pallidum, hippocampus,
	amygdala, accumbens area, ventralDC, choroid-plexus
Func	Left and right cerebral white matter, cerebral cortex, inf
	lateral ventricle, cerebellum white matter, cerebellum cortex,
	thalamus, caudate, putamen, pallidum, hippocampus,
	amygdala, accumbens area, choroid-plexus
Potato	Left and right lateral ventricle, cerebral
	white matter, thalamus, caudate, putamen, pallidum,
	hippocampus, amygdala

### Surface connectivity marker, procrustes marker, and volume marker

We assume that proximity may reflect aspects of functional brain connectivity and have therefore looked at both the individual regions positional relationship, and how they relate to each other. We have calculated the percentage of how much of a regions own surface was connected to the surface of all other regions resulting in a surface connectivity marker. This was done non-symmetric in a voxel-count based manner on the three dimensional data so we had a unique image of each region where zero means that there was no connections between the regions and an increasing percentage number referred to how much surface connectivity existed. This way we could observe if shrinkage of regions relates to more fluid in between regions or general shrinkage where the relative sizes did not change.

The individual regions and their internally relationship was investigated as a change in position of the individual region. We calculated the center of mass *c*∈**R** for each region *P* as the mean position of all the points inside the regions in all of the coordinate directions:

(1) $$c\cdot e_{d}=\frac{1}{2V}\overset{N-1}{\underset{i=0}{\sum }}\int_{A_{i}}{\left(x\cdot e_{d}\right)}^{2}(n_{i}\cdot e_{d}),\text{d =1,2,3}$$

where *e*_*d*_ denote the standard basis in **R** by {*e*_1_,*e*_2_,*e*_3_} and *V* denote the volume. These points were aligned with a Procrustes alignment, where they were adjusted to be in the same space by translation, rotation and scaling of the points [39]. We used the mean shape as the starting shape. This resulted in a feature vector in a machine learning setting that was able to describe the variations in the points related to the disease status.

For comparison we have used the volume measurement for the whole brain and for hippocampus, for which good classification results earlier have been reported [18,19,40]. The whole brain volume fraction included all regions in the skull-stripped brain except for vessels and CSF divided with the intracranial volume. The hippocampus volume fraction was also measured as the lateral hippocampus volume divided with the intracranial volume. A summary of our markers is shown in Table 3.

**Table 3:** An overview of the names and description of the markers we used in this paper

**Marker**	**Description**
Procrustes	The center of mass of each regions aligned
	to the same space with a Procrustes alignment.
Surface connectivity	The percentage of how much each region
	have connected to other regions related
	to the surface of the region.
Hippocampus volume	The volume of the hippocampus divided
	with the intracranial volume.
Whole brain volume	The volume of the whole brain divided
	with the intracranial volume.

### Dimensionality reduction and classification

We wanted to reduce the number of parameters in the case of Procrustes and surface connectivity due to the curse of dimensionality where we had more parameters than observations. We wanted to maintain the relationship between the predictive and target parameters and have therefore chosen to do dimensionality reduction using partial least square regression (PLS) [41]. The idea behind PLS is to find the relevant variables *X* that accounts for as much information of the data *Y* as possible. PLS searches for the set of components (latent variables) that performs a simulation decomposition of *X* and *Y* with the constraint that these components should explain as much as possible of the covariance between *X* and *Y*. It is followed by a linear regression step where the decomposition of *X* is used to predict *Y*. The PLS model will try to find the multidimensionality direction in the *X* space that explains the maximum multidimensional variance direction in the *Y* space. The number of PLS components were set to 10 based on our training experiments. Due to its simple functionality we have used linear discriminate analysis (LDA) for the classification [42]. LDA tries to reduce the dimensionality while preserving as much of the class discriminatory information as possible. LDA seeks to obtain a scalar *y* by projecting the samples *x* onto a line *y*=*w*^*T*^*x* where *x* is the samples and *w* contains the class information. Of all possible ways to discriminate these we would like to select the one that maximizes the separability between the scalars *y*.

All experiments were done in a leave-one-of-each-class out fashion. The data were adjusted for age and gender when there existed a linear correlation between those.

## Result

The fractional volume scores for the whole brain volume and hippocampus volume for NC, MCI, and AD, respectively is shown in Table 4. NC had a larger volume in both whole brain and hippocampus than MCI and AD, and MCI had a larger volume score than AD. AD had the largest volume lost between bl and m12.

**Table 4:** Fractional volume scores for the hippocampus and the whole brain at bl and month 12, and the volume loss

**Group**	**Time point**	**Whole brain**	**Hippocampus**
		**volume fraction (cm**^**3**^**)**	**volume fraction (cm**^**3**^**)**
NC	bl	0.6139 (±0.0451)	0.0045 (±6.6958e-004)
*n*=170	month 12	0.6087 (±0.0465)	0.0044 (±7.0889e-004)
	delta	0.0050 (±0.0146)	9.7840e-005 (±3.1796e-004)
MCI	bl	0.5908 (±0.0398)	0.0038 (±6.7920e-004)
*n*=240	month12	0.5815 (±0.0422)	0.0037 (±6.8807e-004)
	delta	0.0084 (±0.0155)	1.4248e-004 (±2.5027e-004)
AD	bl	0.5769 (±0.0410)	0.0035 (±6.2344e-004)
*n*=114	month12	0.5666 (±0.0402)	0.0033 (±5.9287e-004)
	delta	0.0106 (±0.0136)	1.6425e-004 (±2.6376e-004)

All scores were normalized by the intracranial volume. NC had the larges volume scores and AD had the largest volume loss.

**Figure 2:**
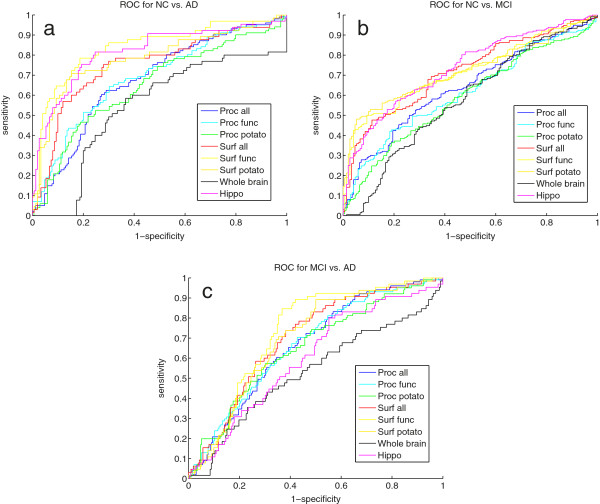
(a) show the ROC for AD vs NC, (b) shows the ROC for NC vs. MCI, and (c) shows the ROC for MCI vs. AD.

For each feature set the area under the curve (AUC) was computed and summarized in Table 5 for NC versus AD, NC versus MCI, and MCI versus AD and the corresponding ROC curves are shown in Figure 2. The classification was tested with a ranksum test and the p-values are also shown in Table 5. All markers were able to significantly discriminate between the three groups NC-AD, NC-MCI, and MCI-AD. The AUC score were highest for the NC-AD group, where our surface connectivity marker were comparable to the hippocampus volume for the AD-NC and NC-MCI cases and better in the discrimination for the MCI-AD case than the hippocampus volume. The AUC for the Procrustes marker were in general a little lower than for the surface connectivity score.

**Table 5:** The AUC values and corresponding ranksum p-values for classification of AD-NC, NC-MCI, and MCI-AD

**(a) Baseline data, not adjusted**						
	NC-AD AUC	p −value	NC-MCI AUC	p −value	MCI-AD AUC	p −value
HP/ICV	0.878	<0.001	0.783	<0.001	0.635	<0.001
WB/ICV	0.724	<0.001	0.648	<0.001	0.648	<0.001
Surface all	0.818	<0.001	0.765	<0.001	0.740	<0.001
Surface func	0.877	<0.001	0.785	<0.001	0.766	<0.001
Surface potato	0.849	<0.001	0.785	<0.001	0.736	<0.001
Procrustes all	0.769	<0.001	0.679	<0.001	0.707	<0.001
Procrustes func	0.784	<0.001	0.656	<0.001	0.712	<0.001
Procrustes potato	0.752	<0.001	0.640	<0.001	0.705	<0.001
**(b) Baseline whole brain, bl. volume adjusted**						
	NC-AD AUC	p −value	NC-MCI AUC	p −value	MCI-AD AUC	p −value
Surface all	0.752	<0.001	0.664	<0.001	0.574	0.024
Surface func	0.839	<0.001	0.695	<0.001	0.597	0.006
Surface potato	0.787	<0.001	0.705	<0.001	0.600	0.003
Procrustes all	0.678	<0.001	0.566	0.001	0.520	0.022
Procrustes func	0.689	<0.001	0.539	0.006	0.572	<0.001
Procrustes potato	0.650	<0.001	0.513	0.010	0.582	<0.001
**(c) Baseline hippocampus volume, bl. volume adjusted**						
	NC-AD AUC	p −value	NC-MCI AUC	p −value	MCI-AD AUC	p −value
Surf all	0.639	0.001	0.608	<0.001	0.688	<0.001
Surf nfunc	0.739	<0.001	0.615	<0.001	0.729	<0.001
Surf potato	0.667	<0.001	0.622	<0.001	0.671	<0.001
Procrustes all	0.624	0.001	0.575	0.010	0.663	<0.001
Procrustes nfunc	0.631	<0.001	0.553	0.068	0.671	<0.001
Procrustes potato	0.574	0.041	0.529	0.328	0.658	<0.001

The last two markers were divided in three groups all, functional, and potato-shaped. 5(a) is the non-adjusted case, 5(b) and 5(c) is adjusted by whole brain fraction and hippocampus fraction, respectively. All markers were able to significantly distinguish the classes. Our markers were still significant after adjustment for the two volume scores, but AUC scores were in general lower than the non-adjusted scores. The surface connectivity score for the functional groups performed the best.

Next, we adjusted our markers for whole brain volume and for hippocampus volume to investigate if our markers contained additional information than the volumes. These results are shown in Table 5. The signal lowers but was still significant. Again, the surface connectivity markers performed better then the Procrustes markers and the NC-AD classification result were the best. The surface connectivity markers were generally better to discriminate NC-MCI than MCI-AD, and for the Procrustes markers it was vice versa. It was the smaller groupings; functional and potato-shaped, that gave the best performance.

**Figure 3:**
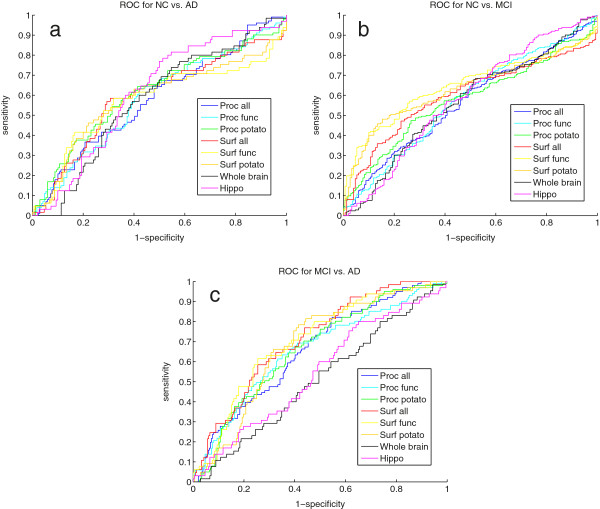
(a) show the ROC for AD vs NC, (b) shows the ROC for NC vs. MCI, and (c) shows the ROC for MCI vs. AD.

We have also investigated how our markers performed on the period to month 12 using the score differences between bl and month 12 for each marker, and the AUC and the corresponding ranksum p-values are shown in Table 6 and roc curves in Figure 3. Hippocampus and whole brain showed relatively low AUC result due to the use of static Freesurfer volumes from bl and month 12. Our surface connectivity scores performed the best for all three groups NC-AD, NC-MCI, and MCI-AD. The results between NC-AD and NC-MCI are very similar.

**Table 6:** Classification result for NC-AD, NC-MCI, and MCI-AD for the difference between the bl and month 12 makers 6(a) is the not adjusted case, 6(b) is adjusted for bl whole brain volume and 6(c) is adjusted for baseline hippocampus volume

**(a) Delta values, not adjusted**						
	NC-AD AUC	p −value	NC-MCI AUC	p −value	MCI-AD AUC	p −value
HP/ICV	0.579	0.068	0.567	0.030	0.526	0.030
WB/ICV	0.600	0.020	0.588	0.004	0.588	0.004
Surface all	0.664	<0.001	0.643	<0.001	0.719	<0.001
Surface func	0.729	<0.001	0.732	<0.001	0.736	<0.001
Surface potato	0.716	<0.001	0.717	<0.001	0.718	<0.001
Procrustes all	0.630	<0.001	0.591	0.002	0.672	<0.001
Procrustes func	0.636	<0.001	0.612	<0.001	0.676	<0.001
Procrustes potato	0.695	<0.001	0.626	<0.001	0.681	<0.001
**(b) Whole brain, bl. volume adjusted**						
	NC-AD AUC	p −value	NC-MCI AUC	p −value	MCI-AD AUC	p −value
Surface all	0.629	0.003	0.630	<0.001	0.725	<0.001
Surface func	0.657	0.000	0.704	<0.001	0.739	<0.001
Surface potato	0.645	0.001	0.681	<0.001	0.707	<0.001
Procrustes all	0.605	0.004	0.575	0.011	0.655	<0.001
Procrustes func	0.593	0.011	0.586	0.003	0.647	<0.001
Procrustes potato	0.640	0.000	0.600	0.001	0.657	<0.001
**(c) Hippocampus volume, bl. volume adjusted**						
	NC-AD AUC	p −value	NC-MCI AUC	p −value	MCI-AD AUC	p −value
Surface all	0.591	0.034	0.597	0.002	0.712	<0.001
Surface func	0.575	0.082	0.649	<0.001	0.704	<0.001
Surface potato	0.582	0.056	0.630	<0.001	0.681	<0.001
Procrustes all	0.580	0.028	0.564	0.028	0.659	<0.001
Procrustes func	0.583	0.022	0.573	0.013	0.657	<0.001
Procrustes potato	0.615	0.002	0.577	0.008	0.664	<0.001

Our markers was still able to significantly discriminate between the three groups. Our surface connectivity markers for the two subgroups functional and potato performed the best.

We have adjusted the month 12 classification results for both the baseline whole brain and the baseline hippocampus volume shown in Table 6. The results showed a significant classification for our markers. When adjusted for whole brain volume the surface connectivity performed the best. The classification result for MCI-AD case was better than the NC-AD result.

Finally, we have classified MCI-c against MCI-nc, where the non-adjusted result is shown in Table 7. The surface connectivity markers was the only marker that was able to distinguish the two groups and only in the functional and potato-shaped grouping of regions. When we adjusted for whole brain volume the surface connectivity marker was still significant with an AUC at 0.631 (*p*=0.012) and for the potato group it was borderline significant with an AUC at 0.595 (*p*=0.067). In the case where we adjusted for hippocampus volume, only the surface connectivity marker for the functional groups was borderline significant with an AUC of 0.599 (*p*=0.055). No other significance were shown in the adjusted cases.

**Table 7:** The AUC and corresponding p-values for the classification of MCI-c and MCI-nc

**Markers**	**AUC**	**p −value**
HP/ICV	0.466	0.516
WB/ICV	0.512	0.823
Surface all	0.542	0.416
Surface func	0.624	0.017
Surface potato	0.603	0.048
Procrustes all	0.465	0.486
Procrustes func	0.498	0.964
Procrustes potato	0.534	0.501

Only the surface connectivity markers was able to significantly discriminate the two groups; functional and potato-shaped.

## Discussion and conclusion

We have investigated a novel way of looking at the relationship between different regions in the brain. We evaluated a surface connectivity marker and center of mass based marker and their ability to classify between NC, MCI, and AD subjects. Both markers have been able to significantly discriminate between the three classes AD-NC, NC-MCI, and MCI-AD both at baseline and between baseline and month 12. Our surface connectivity marker was also able to classify MCI-c.

The large variability’s in the brain regions is related to Alzheimer’s Disease [17,19,25-27], and this have motivated our two markers describing the proximity between the regions in the brain. Both our markers were able to significantly differentiate between AD and NC, also when adjusted for whole brain and hippocampus volume. The surface connectivity marker was comparable to hippocampus volume, which is one of known most effect full markers from MRI. Also after adjustment for volumes we had a significant classification results, this indicates that our markers hold additional information about the development of the brain in relation to progression of AD. We believe that our markers capture an individual shrinkage due to pathological alterations. In subjects with AD the cerebral cortex is shrinking, the sulci’s is widened, the cortical ribbon may be thinned and ventricles are dilated [2,43,44]. Our surface connectivity markers may capture some of these pathological alterations in measuring the proximity between regions.

We have evaluated our markers over a 1 - year period where we have investigated the change in the Procrustes aligned positions and the change in surface connectivity. In this case, we were also able to significantly discriminate between the classes, although the signal was less strong. The weakened signal can be due to noise in the segmentation of the data. Our markers were not taken from registered brains but normalized within the same brain so they captured comparable information across time and study population. The segmentation of the individual regions at two time steps can still be quite different and when we were using the difference between the score values it can introduce noise in our markers. This is also visible in the values for hippocampus and whole brain volume in the longitudinal part of our study, which showed lower results for classification than other reported results [17,45].

Our surface connectivity marker performed the best indicating that it captured how the cell death caused by AD minimizes the surface connectivity between regions. This was most visible in the functional regions. The functional group were limited to functional regions of the brain and the good performance of this grouping is in line with the knowledge that AD affect the network around and including the medial temporal lobe and disruption in this region contributes to memory impairment [46]. The lower performance of our Procrustes marker could be due to the captured information is closer to volume and that no particular regions moves related to the others, but all regions moved due to general volume loss.

Cuingnet et al. [18] have made a comparison study for classification of NC versus AD, NC versus MCI-converters (MCI-c), and MCI-c versus MCI-nonconverters (MCI-nc) based on 81 NC, 67 MCI-nc, 39 MCI-c, and 69 AD subjects from the ADNI database. They investigated voxel based segmented tissue regions for the whole brain in six different variants and for gray matter (GM) and GM, white matter (WM), and cerebrospinal fluid (CSF) combined, cortical thickness in three different variants, and finally hippocampus volume and shape in three different variants a total of ten different methods. They conclude that all methods were able to classify NC vs. AD with a sensitivity and specificity at the range from 59 *%* - 81 *%* and 77 *%* - 98 *%*, respectively, which is comparable to our classification. Other prediction studies have shown better classification rates at 67 *%* - 92 *%* for cross-sectional studies [14,17,19,45,47] and 69 *%* - 81,5 *%* for longitudinal studies [19-21]. The difference in the classification accuracy between our method and the other papers can be explained by the tuning of methods and the use of different data sets.

Only our surface connectivity marker was able to classify MCI-c from MCI-nc and not with a highly significant result. This is in line with Cuignet et. al comparison study for AD classification, where they found that only four methods managed to predict MCI-c vs MCI-nc better than a random classifier and none of those got significantly better results [18]. The main reason for the low result in the conversion case could be due to the fact that MCI is a very in heterogeneous group that possibly could convert rapidly to AD or be stable for many years before conversion.

Other studies have investigated the change locally in the hippocampus. Wang et al. [13] have used large-deformation diffeomorphic high-dimensional brain mapping to quantify and compare changes in the hippocampal shape as well as volume. They found that shape changes were largely confined to the head of hippocampus and subiculum for normal controls (NC). Other studies have confirmed these shape changes for the hippocampus [14-16] based on shape models and local hippocampal atrophy patterns. We have focused on investigating the relationship between the different regions of the brain and how they differ between healthy subjects and AD patients. This way of investigating the regions could make it possible to incorporate different kind of knowledge into the same model where one could go from the individual scale of each region, to the interaction between the regions and finally to combined picture of the brain as one whole region.

An alternative use of MRI images for early prediction of AD is by using texture analysis where different textures features is used to construct a computational framework which have been able to discriminate AD, MCI and NC with a separability of up to 95 *%*[23,40,48]. This indicates that one can combine the three different kinds of markers; volume, texture and shape/proximity markers to get a more sophisticated picture of the disease progression.

Other image modalities such as single-photon emission computed tomography (SPECT), functional MRI and MR spectroscopy (MRS), positron emission tomography (PET), and molecular imaging have been used for investigation of brain changes related to AD. SPECT combined with MRI images can give additional information about disease progression when combined [49]. Functional MRI and MR spectroscopy (MRS) have shown changes in metabolic levels even prior to symptom onset in AD, but are difficult to implement in clinical settings due to technical support [50,51]. PET metabolic imaging with radioactive glucose has also been used to examined the functional change and tracking of the AD disease progression [52,53]. Due to the invasiveness, radiation dose limitation, requiring lumbar punctures and high cost, PET is unsuitable for repeated measurements of a single patient or screening programs for large populations. Molecular imaging with amyloid tracers have showed great potential as to be accurate markers for early diagnosis of AD, but do not show progression in established disease [54,55], which is our object of interest.

To conclude structural MRI is an suitable image modality for detection of AD and AD progression. Our markers have shown promising results in capturing how the proximity of different regions in the brain can aid in AD diagnosis and prognosis. The proximity analysis captures additional information about the whole brain compared to atrophy scores. This additional information can contribute to the refinement of the AD markers and may be able to give a more detailed picture of AD progression.

## Competing interests

The authors declare that they have no competing interests.

## Authors’ contributions

LL have contributed in study design, data analysis and interpretation, prepared and submitted the manuscript. LS and AP performed study design and data collection. EBD and MN participated in design and reviewed manuscript. All authors have read and approved the final manuscript.

## Pre-publication history

The pre-publication history for this paper can be accessed here:

http://www.biomedcentral.com/1471-2342/14/21/prepub
